# The Role of Artificial Intelligence in the Diagnosis of Melanoma

**DOI:** 10.7759/cureus.69818

**Published:** 2024-09-20

**Authors:** Sadhana Kalidindi

**Affiliations:** 1 Clinical Research, Apollo Radiology International Academy, Hyderabad, IND

**Keywords:** convolutional neural networks, dermoscopy, artificial intelligence, machine learning, melanoma

## Abstract

The incidence of melanoma, the most aggressive form of skin cancer, continues to rise globally, particularly among fair-skinned populations (type I and II). Early detection is crucial for improving patient outcomes, and recent advancements in artificial intelligence (AI) have shown promise in enhancing the accuracy and efficiency of melanoma diagnosis and management.

This review examines the role of AI in skin lesion diagnostics, highlighting two main approaches: machine learning, particularly convolutional neural networks (CNNs), and expert systems. AI techniques have demonstrated high accuracy in classifying dermoscopic images, often matching or surpassing dermatologists’ performance. Integrating AI into dermatology has improved tasks, such as lesion classification, segmentation, and risk prediction, facilitating earlier and more accurate interventions.

Despite these advancements, challenges remain, including biases in training data, interpretability issues, and integration of AI into clinical workflows. Ensuring diverse data representation and maintaining high standards of image quality are essential for reliable AI performance. Future directions involve the development of more sophisticated models, such as vision-language and multimodal models, and federated learning to address data privacy and generalizability concerns. Continuous validation and ethical integration of AI into clinical practice are vital for realizing its full potential for improving melanoma diagnosis and patient care.

## Introduction and background

Skin cancer is one of the most prevalent forms of cancer and is known to have a high malignancy rate. In fair-skinned individuals, its incidence has increased annually by 3-7% over the past few decades, surpassing the incidence of almost all other malignancies [1,2]. Therefore, thorough screening programs and tests are crucial to identify skin cancer in its early stages. Automating the identification of skin moles using computational artificial intelligence (AI) and image analysis techniques is essential for achieving this goal. The most serious form of skin cancer, melanoma, can be identified early, and this has a major impact on patient outcomes. It reduces the chance of life-threatening consequences, slows the progression of the disease, and increases the possibility that therapy will be successful. In addition, shorter recovery times, less invasive surgical procedures, and successful use of adjuvant medicines are all made possible by early identification. Improving patient outcomes mostly depends on timely action and routine skin examinations [3].

AI has become a transformative tool in multiple industries, including medical diagnostics [4-6]. In dermatology, AI has garnered significant interest for its potential to automate the diagnosis and management of skin conditions [7]. Accurate and timely identification of skin lesions, such as melanoma and other skin cancers, is critical for early intervention and improved patient outcomes. Two primary AI methodologies have been employed for skin lesion diagnostics. The first method leverages machine learning (ML) [8], particularly neural networks and various convolutional neural networks (CNNs) [9], which require large and dependable training datasets that are characterized by complex internal mechanisms that are not easily interpretable by human experts. This lack of transparency, often referred to as the 'black-box' problem, arises when machine learning models, particularly deep learning (DL) algorithms, process vast amounts of data and generate predictions without providing clear explanations of how specific inputs lead to particular outcomes. As a result, human experts face challenges in validating, trusting, or understanding the decision-making process, limiting the system’s applicability in critical fields like healthcare or legal decision-making where interpretability is crucial [9]. The second method involves expert systems that rely on information ontologies and utilize clear rules and dependencies to codify expert knowledge [10]. While these systems provide transparency and ease of interpretation, they are labor-intensive to develop and maintain.

The incorporation of AI techniques in dermatology has resulted in notable progress in the automated analysis of skin lesion images. These AI algorithms have demonstrated considerable success in various tasks, such as classifying skin lesions, segmenting images, extracting features, and predicting risks. Despite these achievements, recent studies have identified several challenges and obstacles to the application of AI in skin cancer diagnostics [11,12]. This review aims to explore the latest advancements in AI for diagnosing and managing melanoma while also addressing the current issues and potential future developments in this field.

## Review

Epidemiology of melanoma

Although the incidence and mortality rates of most cancers have been declining over the years, melanoma continues to increase, particularly among fair-skinned populations of European descent. The global melanoma burden has increased by 41% from 230,000 cases in 2012 to 325,000 in 2020, with the highest rates observed in Australia and New Zealand [13]. In the United States (US), melanoma is the fifth most commonly diagnosed cancer, with an estimated 99,780 new cases and 7,650 deaths in 2022 and 97,920 new cases of melanoma in situ, closely matching the number of invasive melanoma cases [14]. Invasive melanomas represent only 1% of all skin cancer cases but are responsible for over 75% of skin cancer deaths, with a five-year survival rate of 93.5%, primarily because of a high proportion of localized disease [15]. Despite the high survival rate, most melanoma deaths are due to Stage I disease progression, although survival rates for Stage III and IV diseases have improved significantly to 73.9% and 35.1%, respectively, owing to advancements in targeted therapies and immunotherapy [15,16]. The median age of melanoma diagnosis in the US is 66 years, with lifetime risks of 2.6% for white people, 0.6% for Hispanic people, and 0.1% for black people [17]. Men are 1.6 times more likely to develop melanoma than women, with the common sites being the arms and legs for women and the head, neck, back, and trunk for men [18]. African American patients are more prone to developing melanoma in sun-protected areas and generally present with more advanced disease at diagnosis, leading to a poorer prognosis compared to Caucasian patients [15].

Traditional diagnostic modalities

*Classification System*****

The current clinicopathological classification of melanoma categorizes melanomas into four main subtypes: lentigo maligna, superficial spreading, acral lentiginous, and nodular, each with specific in situ lesions [19,20]. This classification relies heavily on interpreting histopathological findings, which can be subjective and vary among observers [21-24]. While certain melanomas fit neatly into a subtype, many exhibit mixed histopathologic features, making precise classification challenging [25,26]. For example, acral lentiginous melanomas can display histopathological patterns that are typical of other subtypes. Additionally, this system does not include prognostic data by default; instead, it incorporates extrinsic factors, such as Breslow depth and ulceration. The classification assumes a linear progression model, where in situ lesions precede invasive melanoma and all subtypes are managed based on thickness. Recently, the World Health Organization (WHO) introduced a new classification that adds epidemiologic and genomic information, expanding to nine subtypes, including rare and mucosal melanomas [27]. This updated classification also followed a linear progression model, suggesting that melanocytic nevi with the same driver mutations act as precursor lesions. However, there is scant evidence supporting this model, and the transformation rate of these nevi into melanoma is unknown, with the rare occurrence of such nevi complicating verification. The long-standing study of Clark/dysplastic nevus does not corroborate the precursor model of melanoma progression [28,29].

Clinical Diagnosis

The ABCDE criteria, including asymmetry, border irregularity, color variability, diameter of 6 mm or more, and evolution, have enhanced melanoma diagnosis sensitivity, with a naked-eye examination accuracy of approximately 65% [30-32]. Dermatoscopy, which uses 10× magnification and polarized light, has significantly improved bedside melanoma diagnosis by revealing subsurface structures and developing diagnostic criteria for various skin diseases [33]. Algorithms such as the seven-point checklist, Menzies method, and color, architecture, symmetry, and homogeneity (CASH) criteria have improved melanoma identification sensitivity and specificity by up to 18% and 10%, respectively [34-37]. Despite these improvements, a recent Cochrane review pointed out limited evidence for the effectiveness of dermatoscopy, stating that its practical utility remains unconfirmed [38]. Additionally, the pigmented lesion assay, a tape-strip test that assesses ribonucleic acid (RNA) markers overexpressed in melanoma, has demonstrated over 99% negative predictive value and over 91% sensitivity, though further validation is needed in general clinical practice [39-41].

*Histopathological and Molecular Diagnosis* 

Histopathological diagnosis of melanoma remains the gold standard, yet it is subjective and marked by interobserver variability, especially with smaller, thinner lesions [21-24]. Advances in immunohistochemical staining, such as PReferentially expressed Antigen in MElanoma (PRAME) and p16, have enhanced diagnostic accuracy through molecular insights [41-45]. In parallel, molecular diagnostics such as comparative genomic hybridization (CGH) and fluorescence in situ hybridization (FISH) offer greater precision. CGH detects chromosomal copy number variations with over 95% sensitivity in melanomas, while FISH, which targets specific genomic segments, provides high sensitivity and specificity, albeit with some false positives in polyploid lesions such as Spitz nevi [46-48]. Gene expression profiling (GEP) tests, such as myPath, leverage real-time quantitative reverse transcription polymerase chain reaction (qRT-PCR) technology to assess gene expression and show high accuracy in clear melanocytic lesions, although more studies are needed for ambiguous cases [49-51].

Dermoscopy

Dermoscopy, also called dermatoscopy or epiluminescence microscopy, is a noninvasive method that uses a handheld dermatoscope to examine skin lesions [52,53]. This technique allows for enhanced visualization of the superficial layers of the skin, leading to better diagnostic accuracy for pigmented lesions compared with the naked eye [54,55]. The procedure starts by distinguishing melanocytic from non-melanocytic lesions, followed by classification of the melanocytic lesion with an algorithm. Adding dermoscopy to melanoma screening reduces the number of excisional biopsies performed [56]. Diagnostic accuracy depends on the dermatologist's expertise, the complexity of the lesions, and the assessment algorithm used, with experts performing better than non-experts [57-59]. A systematic review of studies from 1987 to 2000 concluded that dermoscopy enhanced melanoma diagnostic accuracy for experienced practitioners [52]. Nonetheless, dermoscopy does not always definitively differentiate early melanomas from benign melanocytic lesions [52].

AI principles

AI's applications of AI are vast, particularly in medicine, where it improves healthcare delivery, clinical decision-making, and patient treatment planning [60-62]. ML is considered the core of AI and includes supervised, unsupervised, and reinforcement learning algorithms, each with distinct learning methods [63,64]. Deep learning (DL), an advanced ML type using multilayer neural networks, has become crucial in medical applications, predicting drug responses, and optimizing dosages through complex data analysis [65,66]. AI's role in oncology is significant. It enhances clinical image interpretation and diagnoses, as exemplified by FDA-approved software for early cancer detection [67-69]. In melanoma, AI aids precision medicine by analyzing genetic, molecular, and biochemical data, tailoring treatments, and predicting therapeutic efficacy [70-73].

The role of AI in skin pathology

*AI and Diagnosis of Pigmented Lesions*****

Pigmented lesion classification initially utilized classical ML techniques in the 1990s, with CNN-based methods emerging in 2016 [74-76]. These techniques often involve preprocessing to reduce illumination and noise, and to smooth the surrounding skin texture to enhance lesion interpretation. Nasr-Esfahani et al. created a CNN model that was trained on a dataset expanded from 170 to 6120 images through cropping, scaling, and rotation. This model achieved 81% sensitivity, 80% specificity, and 81% accuracy for melanoma detection [74]. Other studies reported sensitivities up to 90% and accuracies between 82% and 94% [76-78]. Esteva et al. showed that a pre-trained CNN using 129,450 images, including 3,374 dermoscopy images, matched the performance of 21 board-certified dermatologists, with an area under the curve (AUC) of 0.96 for carcinomas and melanomas [79].

Soenksen et al. explored AI's accuracy in classifying skin lesions from wide-field images, achieving 90% sensitivity and specificity in distinguishing suspicious lesions [80]. Their study also highlighted an 83% agreement between CNN's intrapatient saliency ranking and dermatologists' rankings. Although dermoscopic images are more frequently used in AI studies, a review indicated that only 12 of 51 studies used gross images, stressing the need for more research with clinical images [81]. Given that dermoscopy is often inaccessible to non-dermatologists, further examination of gross clinical images is essential [82-85]. 

Chatbots in dermatoscopy serve as AI-driven tools designed to assist dermatologists by generating differential diagnoses and educational insights based on dermoscopic descriptions of skin lesions, enhancing diagnostic accuracy and supporting novice practitioners. A recent survey showed that AI Chatbots performed well in generating differential diagnoses for basal cell carcinoma; however, its performance was lower for squamous cell carcinoma and inflammatory dermatoses, though participants remained generally satisfied with its diagnostic and educational capabilities [86].

*AI and Pigmented Lesion Dermoscopy* 

AI has been used in dermoscopic image analysis for over 20 years, initially utilizing classical ML techniques, before transitioning to CNNs in 2016. These advancements have enabled a more accurate classification of dermoscopic images as benign or malignant [74,76,77]. Comparative studies have shown that AI can match or even outperform human experts in the identification of pigmented skin lesions. For example, Esteva et al. found that a pre-trained CNN achieved an area under the curve (AUC) of 0.96 for both carcinomas and melanomas, surpassing the performance of dermatologists in certain scenarios [79]. Other studies have similarly reported higher specificity and sensitivity for CNNs than for dermatologists when given clinical and dermoscopic data [87].

Research has also explored how AI can aid dermoscopic evaluation, particularly in low-confidence diagnoses. Marchetti et al. showed that AI support increased the accuracy of lesion classification for dermatologists and residents, increasing correct classification rates from 73.4% to 75.4% and from 69.4% to 72.6%, respectively [88]. However, AI models are sensitive to minor image changes that do not affect human examiners, as demonstrated by Maron et al., in which slight image alterations led to significant diagnostic variability [89].

CNNs have been applied to classify pigmented lesions in special locations, such as acral and mucosal surfaces, with mixed results. Winkler et al. found high accuracy for lentigo maligna melanoma (AUC of 0.926), acral melanoma (0.928), and superficial spreading melanoma (0.989) but lower accuracy for mucosal and nail unit melanomas (AUCs of 0.754 and 0.621, respectively) [90]. Comparisons between CNNs and dermatologists show that CNNs can perform as well as or sometimes better than experienced dermatologists [91]. Furthermore, AI has improved clinical decision-making, and Lee et al. demonstrated that CNN-generated diagnoses enhanced clinicians' accuracy in evaluating acral-pigmented lesions, improving concordance and reducing performance disparities among different physician groups [92].

*AI and Pigmented Lesion Pathology* 

Computer-aided histopathologic diagnosis began with the introduction of TEGUMENT in 1987, which used a decision tree to assist pathologists, although it saw limited use due to its oversimplification of medical knowledge [93]. A renewed interest in AI for dermatopathology came with whole-slide image (WSI) scanners and CNNs, facilitating advanced image classification [94]. In 2019, Hekler et al. used CNNs to classify melanocytic lesions, achieving a 19% discordance rate with dermatopathologists, comparable to the pathologists' own variability [95]. Hart et al. demonstrated the critical role of image selection in CNN training for classifying melanocytic neoplasms into Spitz and conventional melanocytic nevi. The CNN achieved 92% accuracy with curated images but only 52% accuracy with non-curated images owing to misclassification based on predominant features [96]. This study highlights the need for meticulous image selection to maintain high diagnostic accuracy in AI models.

AI faces challenges in pathology owing to the immense variability in histologic morphology, necessitating large training datasets. Brinker et al. addressed this using a larger comparator group of dermatopathologists, achieving a CNN accuracy of 92% with annotated slides and 88% with unannotated slides [97]. AI models are still sensitive to slide staining variations and are limited by binary classification schemes, which can oversimplify complex diagnoses, such as neoplasms with uncertain malignant potential.

AI and dermatopathology education

Historically, the lack of frameworks for organizing medical knowledge has hindered the development of AI in education. Feit et al. created the Hypertext Atlas of Dermatopathology, an online resource containing over 3,000 high-resolution images with annotations and clinical and microscopic descriptions. This resource allows users to narrow or broaden differential diagnoses through hypertext links, providing detailed information upon reaching a target diagnosis. However, its utility depends on the user's dermatopathology knowledge base [98]. Crowley et al. developed SlideTutor, an intelligent tutoring system with a virtual microscope, and WSIs that teach algorithmic problem-solving in the visual classification of inflammatory dermatoses. This system provides step-by-step feedback, simulating one-on-one tutoring and improving diagnostic reasoning by guiding learners through morphological feature identification, differential diagnosis, and final diagnosis [99-101]. In a study comparing case- and knowledge-focused interfaces in SlideTutor, both interfaces demonstrated equivalent learning gains and retention, although students preferred the knowledge-focused interface because of their higher certainty levels [102]. AI-based systems such as SlideTutor can measure diagnostic errors and heuristics, providing valuable feedback to reduce errors and enhance learning [103,104].

AI has also shown potential for diagnostic reporting education. Crowley et al. developed ReportTutor, an intelligent tutoring system combining a virtual microscope, WSIs, and a natural language interface to aid dermatopathology trainees in preparing diagnostic reports for melanoma. This system provides feedback on prognostic features, measurement accuracy, and report style, thereby promoting standardized and accurate diagnostic reports [105]. El Saadawi et al. evaluated the impact of feedback timing on AI-based instructions for melanoma diagnostic reporting. Both immediate and delayed feedback led to significant learning gains, with most improvements occurring early in the tutoring sessions. The study also explored metacognition and found no correlation between learning gains and learners' certainty about their performance, suggesting that AI-based instruction can effectively enhance diagnostic reporting, regardless of feedback timing [104].

Smartphone apps

With advanced cameras and computational capabilities, smartphones are increasingly being used in medical applications, including skin self-examination and teledermatology. Devices like DermLite and MoleScope improve image quality, enabling patients to monitor lesions and send high-quality images for professional analysis. Early mobile apps for melanoma detection used methods such as ABCD feature extraction and support vector machine (SVM) classifiers. Recent innovations have introduced CNNs for more precise melanoma classification. Iowa State University developed an app using a detachable 10x lens, achieving 88% accuracy with a support vector machine (SVM) classifier based on a radial basis function (RBF) kernel [106]. Another application, using CNN, achieved 78.8% accuracy, 91.3% sensitivity, and 73% specificity on 8,000 images [107]. The PAD-UFES-20 dataset app leveraging a modified ResNet50 CNN achieved 85% accuracy and 96% reproducibility by incorporating clinical data [108]. The SkinScreener app, now a commercialized medical device, demonstrated a sensitivity of 96.4% and specificity of 94.85% for melanoma risk assessment [109]. SkinVision, a widely reviewed application, has used ML algorithms since 2018, showing a sensitivity of 95% and specificity of 78% in triaging skin lesions based on over 130,000 training images [110-112].

Limitations and challenges

AI research in dermatology is in its early stages and encounters various challenges, such as biases, interpretability issues, regulatory barriers, and integration difficulties with current clinical workflows. AI algorithms must be robust and transparent to enhance patient care without new complications. AI algorithms learn from training datasets; however, confounders can also affect their accuracy. For example, surgical pen markers on images can mislead AI models by associating them with malignancy, as noted by Winkler et al. [113]. Additionally, biases in training datasets can reinforce existing healthcare disparities, with early AI models underperforming on darker skin types owing to a lack of diverse data representation [114]. It is imperative to ensure diverse and equitable data representations to produce accurate and fair AI output.

Maintaining image quality and standardizing capturing modalities in dermatology AI research is critical for data consistency. Diverse sources and settings create heterogeneous datasets that affect AI performance [115,116]. Techniques such as saliency maps and content-based image retrieval are being developed to improve AI transparency and trust. Integrating AI into clinical practice presents further challenges, including medical-legal issues, data privacy, and liability [117,118]. AI models require continuous validation to maintain reliability, as many have been approved based on retrospective data, without thorough prospective trials [119]. Building trust among stakeholders and validating AI models across multiple sites is crucial for realizing AI's full potential in dermatology [120,121]. Table 1 summarizes the challenges associated with AI in dermatopathology.

**Table 1:** Challenges associated with AI in dermatopathology. AI: artificial intelligence.

Challenge	Summary
Model validation	Many AI models lack true external validation sets, making them less representative of the general population. Furthermore, standardized benchmarks for comparing models are scarce due to the limited availability of comprehensive public datasets.
Data quality	The performance of AI models can be hindered by data quality issues, which may arise from user errors during initial data collection, resulting in artifacts, or from inherent deficiencies in the source data, leading to limited diversity and class imbalances not addressed by the model.
Algorithmic bias and health equity	AI models may exhibit biases due to the training data selection, affecting their ability to generalize across different racial and socioeconomic demographics.
Implementation and user confidence	The acceptance of AI is often restricted by regulatory bodies like the FDA, as well as by clinicians and patients, where mistrust or uncertainty may hinder its adoption.

Future directions

Recent advancements in language models, specifically vision-language models (VLMs) and multimodal models, have shown significant potential in dermatology. These models, such as Skin-GPT4, can interpret clinical skin lesion photographs, offer descriptions and diagnoses, and have applications ranging from patient chatbots to triage tools. By integrating patient demographics, visual data, and genetic information, these models aim to provide comprehensive medical insights, enhancing generalist medical AI capabilities [122-124]. As datasets expand and computational power increases, these models are expected to improve in accuracy, aiding in the diagnosis of complex skin conditions and overall dermatological practice.

Federated learning (FL) addresses data privacy and accessibility issues by allowing AI models to be trained on datasets from multiple institutions without data transfer, maintaining privacy, and improving generalizability. Successful in radiology and oncology, FL holds promise in dermatology, especially in reducing performance disparities in underrepresented skin types [125-127]. Foundation models (FMs) can be locally fine-tuned to enable institutions to create customized models for their specific demographics. However, to fully leverage these technologies, the challenges in data quality, aggregation, and infrastructure must be addressed. Additionally, advancements in AI model architecture and comprehensive evaluation metrics, including clinical value, fairness, and transparency, are essential for the progress of dermatological AI [128]. Medicolegal aspects also need careful consideration, as the use of AI in dermatology may raise concerns about liability in cases of misdiagnosis or delayed diagnosis. Ensuring regulatory compliance, establishing clear accountability, and maintaining transparency in AI-driven decision-making will be crucial to addressing potential legal risks associated with AI integration in clinical practice.

## Conclusions

The integration of AI in dermatology, particularly in the diagnosis and management of melanoma, holds significant promise but also presents several challenges that must be addressed. AI techniques, particularly those utilizing CNNs, have demonstrated high accuracy in classifying dermoscopic images and aid in the detection of melanoma. Mobile applications equipped with advanced imaging capabilities and AI algorithms have further enhanced the ability to monitor and assess skin lesions remotely, thereby improving early detection and patient outcomes. However, the variability in image quality, biases in training datasets, and complexity of integrating AI into existing clinical workflows highlight the need for robust and transparent AI models. Ensuring diverse and equitable data representation and maintaining high image quality standards are critical for the accuracy and fairness of AI outputs in dermatology. Future advancements in AI for melanoma diagnosis are likely to be driven by the development of more sophisticated models, such as VLMs and multimodal models, which can integrate diverse data types for comprehensive medical insights. FL offers a solution to data privacy and accessibility challenges by enabling collaborative model training across institutions without data transfer, thereby enhancing generalizability. Additionally, ongoing improvements in the AI model architecture and evaluation metrics are essential to ensure clinical value, fairness, and transparency. As AI continues to evolve, its successful implementation in dermatology depends on addressing these challenges and fostering trust among stakeholders through continuous validation and ethical integration into clinical practice.
